# Reconstructing cancer genomes from paired-end sequencing data

**DOI:** 10.1186/1471-2105-13-S6-S10

**Published:** 2012-04-19

**Authors:** Layla Oesper, Anna Ritz, Sarah J Aerni, Ryan Drebin, Benjamin J Raphael

**Affiliations:** 1 Department of Computer Science, Brown University, Providence, RI, USA; 2 BioMedical Informatics Program, Stanford University, Stanford, CA, USA; 3 Center for Computational Molecular Biology, Brown University, Providence, RI, USA

## Abstract

**Background:**

A cancer genome is derived from the germline genome through a series of somatic mutations. Somatic structural variants - including duplications, deletions, inversions, translocations, and other rearrangements - result in a cancer genome that is a scrambling of intervals, or "blocks" of the germline genome sequence. We present an efficient algorithm for reconstructing the block organization of a cancer genome from paired-end DNA sequencing data.

**Results:**

By aligning paired reads from a cancer genome - and a matched germline genome, if available - to the human reference genome, we derive: (i) a partition of the reference genome into intervals; (ii) adjacencies between these intervals in the cancer genome; (iii) an estimated copy number for each interval. We formulate the Copy Number and Adjacency Genome Reconstruction Problem of determining the cancer genome as a sequence of the derived intervals that is consistent with the measured adjacencies and copy numbers. We design an efficient algorithm, called Paired-end Reconstruction of Genome Organization (PREGO), to solve this problem by reducing it to an optimization problem on an interval-adjacency graph constructed from the data. The solution to the optimization problem results in an Eulerian graph, containing an alternating Eulerian tour that corresponds to a cancer genome that is consistent with the sequencing data. We apply our algorithm to five ovarian cancer genomes that were sequenced as part of The Cancer Genome Atlas. We identify numerous rearrangements, or structural variants, in these genomes, analyze reciprocal vs. non-reciprocal rearrangements, and identify rearrangements consistent with known mechanisms of duplication such as tandem duplications and breakage/fusion/bridge (B/F/B) cycles.

**Conclusions:**

We demonstrate that PREGO efficiently identifies complex and biologically relevant rearrangements in cancer genome sequencing data. An implementation of the PREGO algorithm is available at http://compbio.cs.brown.edu/software/.

## Introduction

A cancer genome is derived from the germline genome through a series of somatic mutations that accumulate during the lifetime of an individual. These range in size from single nucleotide mutations through larger structural variants (SVs), that duplicate, delete, or rearrange segments of DNA sequence. These structural variants may amplify genes that promote cancer (oncogenes) or delete genes that inhibit cancer development (tumor suppressor genes). In addition, rearrangements such as translocations and inversions may change gene structure or regulation and create novel fusion genes, with or without concomitant changes in copy number [1]. Classic examples are the BCR-ABL fusion gene in chronic myeloid leukemia and the activation of the MYC oncogene in Burkitt's lymphoma via a translocation. Identification of other common structural aberrations is essential for understanding the molecular basis of cancer and for developing cancer-specific diagnostic markers or therapeutics such as Gleevec that targets BCR-ABL [2] or Herceptin that targets ERBB2 amplification [3]. However, many cancer genomes are aneuploid, containing extensive duplicated sequences, and are highly rearranged compared to the germline genomes from which they were derived. The organization of amplified regions in cancer genomes is often highly complex with many high copy amplicons from distant parts of the reference genome co-localized on the cancer genome [4,5]. Estimating the number of copies of these amplicons is extremely difficult. Moreover, determining whether such extensive rearrangements occurred over many cell divisions or nearly simultaneously (e.g. chromothripsis) is difficult [6].

DNA sequencing technologies have improved dramatically over the past decade, and next-generation DNA sequencing technologies now enable the sequencing of large cohorts of cancer genomes [7,8]. However, all present DNA sequencing technologies are limited in the length of DNA sequences they produce with the most affordable technologies producing reads less than 200bp in length. *De novo *assembly of human, or other mammalian genomes, from this data remains a difficult task [9]. This is primarily due to the presence of repeated sequences in these genomes. *De novo *assembly of cancer genomes is an even more daunting problem due to complications of aneuploidy and heterogeneity described above.

Because of these challenges, somatic mutations in cancer genomes are now typically analyzed through a *resequencing *approach that relies on alignment of DNA sequence reads to the human reference genome. Paired-end sequencing technologies that generate paired reads from a longer DNA fragment (or insert) allow the detection of all types of somatic structural variants. Paired end mapping [10,11], or End Sequencing Profiling [12,13], aligns paired reads from a cancer genome to the reference human genome. The distance between the aligned reads is computed. If this *aligned distance *is close to the length of end sequenced fragments, as determined by the distribution of fragment lengths, the aligned pair of reads is referred to as a *concordant pair*. If the aligned distance is far from the expected fragment length (either shorter or longer) or if the orientation of the aligned reads has changed, then the aligned pair is referred to as a *discordant pair*. Clusters of discordant pairs reveal novel adjacencies (or breakpoints) created by somatic structural aberrations [13]. Numerous methods have been developed in the past few years to identify structural variants by paired end mapping [14-18] and [19] review many of the recent techniques for accomplishing this goal. In addition, when the sequencing coverage is high, the number of aligned reads [20] or concordant pairs [21] provides an estimate of the number of copies of segments of the cancer genome.

In this paper we address the problem of reconstructing the organization of the cancer genome(s) present in a cancer DNA sample from the adjacencies and copy number information revealed by the concordant and discordant pairs from a paired-end resequencing approach. We define the Copy Number and Adjacency Genome Reconstruction Problem, a general formulation of the problem which we solve as a convex optimization problem. Our approach adapts and generalizes techniques that have been employed previously in genome assembly [22-24], ancestral genome reconstruction and genome rearrangement analysis in the presence of duplicated genes [25], and prediction of copy number variants [26]. In contrast to these works, we focus on the particular features and challenges of cancer genome reconstruction including a broad class of rearrangements, aneuploidy, heterogeneity, and the availability of an "ancestral" reference genome. We apply our algorithm, called Paired-end Reconstruction of Genome Organization (PREGO), for solving the Copy Number and Adjacency Genome Reconstruction Problem to simulated cancer genome data and to real sequencing data from 5 ovarian cancer genomes from The Cancer Genome Atlas (TCGA). We identify numerous rearrangements, or structural variants, in these genomes, analyze reciprocal vs. non-reciprocal rearrangements, and identify rearrangements consistent with known mechanisms of duplication such as tandem duplications and breakage/fusion/bridge (B/F/B) cycles.

## Methods

### Intervals, adjacencies, and cancer genome reconstruction

Suppose the cancer genome is derived from the germline genome through a series of somatic rearrangements. We perform paired-end DNA sequencing on a cancer DNA sample . We assume that the sample  contains a genome sequence derived from the reference genome through some series of somatic structural rearrangements of blocks of DNA (we are not considering single nucleotide mutations). From the alignments of paired reads to the reference genome, we derive three pieces of information. First, we derive a partition of the reference genome into a sequence of intervals **I **= (*I*_1_, *I*_2_, ..., *I*_*n*_). Each interval *I*_*j *_= [*s*_*j*_, *t*_*j*_] is the DNA segment from the positive strand of the reference genome that starts at coordinate *s*_*j *_and ends at coordinate *t*_*j*_. Since intervals also appear in the opposite direction in a cancer genome (e.g. due to an inversion), we denote by *I*_-*j *_= [*t*_*j*_, *s*_*j*_] the inverted DNA segment. Second, concurrently with the definition of **I**, we derive a set  of novel adjacencies in the cancer genome. Each adjacency (*I*_*j*_, *I*_*k*_) indicates that the end *t*_*j *_of interval *I*_*j *_is adjacent to the start *s*_*k *_of interval *I*_*k *_in the cancer genome. Thus $A\subseteq \left\{\left(I_{j},I_{k}\right)|j,k\in \left\{\pm 1,\pm 2,\ldots ,\pm n\right\}\right\}$. The partition **I **and associated set of adjacencies  are obtained by clustering discordant paired reads whose distance or orientation suggest a rearrangement in the cancer genome [13]. Any existing algorithm can be used to create such input and therefore, the decision about what data to use (i.e. ambiguous reads, split reads, read mapping quality, etc) are part of upstream processing. Third, we derive a read depth vector **r **= (*r*_1_, ..., *r*_*n*_)^*T*^, where *r*_*j *_is the number of (paired) reads that align entirely within interval *I*_*j*_. The read depth vector **r **is obtained by counting concordant pairs in each interval [27].

Our goal is to reconstruct the *block organization *of the cancer genome(s) in the cancer DNA sample  from the interval, adjacency, and copy number information. The block organization corresponds to a sequence *I*_*α*(1)_*I*_*α*(2) _... *I*_*α*(*M*) _of *M *intervals where each *α*(*j*) ∈ {± 1, ..., ± *n*}. We formulate the following problem.

### Copy number and adjacency genome reconstruction problem

*Given an interval vector ***I***, a set *A*of cancer adjacencies, and a read depth vector ***r ***derived from a cancer sample *S*, find the cancer genome(s) that are most consistent with these data.*

The statement of this problem does not quantify "most consistent". Defining such a quantitative measure requires the consideration of several complicating factors. First, the measurements of adjacencies  and the partition **I **that they determine may be incomplete or inaccurate. Second, many cancer genomes are *aneuploid*, meaning that the copy number of many intervals is above and below the diploid number of 2, and thus the read depth vector may not accurately represent the actual copy number of each interval in the cancer genome. Finally, a cancer sample  consists of many tumor cells, and each of these may contain different somatic mutations. However, because most tumors are clonal originating from a single cell, a large fraction of the important somatic mutations will be found in all cells of the cancer sample . In this paper, we assume that the cancer sample  is genetically homogenous so that we need only construct the organization of one rearranged cancer genome. Below, we formulate a specific instance of the Copy Number and Adjacency Genome Reconstruction Problem that considers the case of a single cancer genome with errors in the set  of adjacencies, sequence **I **of intervals, and the copy numbers must be inferred from the read depth vector **r**. We defer the question of heterogeneity to future work. We first consider the case of perfect data.

### Copy number and adjacency genome reconstruction problem: perfect data

We begin with the case that the data is complete and error-free: thus, all cancer adjacencies  are correctly measured, and we have correctly estimated the copy number of each interval from the read depth vector **r**. Also, for ease of exposition, we assume that the reference and cancer genomes each contain a single chromosome. Specifically, we define the *interval count vector ***c **= (c_1_, c_2_, ..., *c*_*n*_)^*T*^, where *c*_*j *_indicates how many times the interval *I*_*j *_occurs in . Note that in general **c **is not directly measured, but rather must be inferred from the data, and we consider this extension in the next section. We have the following problem.

### Single chromosome copy number and adjacency genome reconstruction problem

*Given an interval vector ***I***, a set **of cancer adjacencies, an interval count vector ***c***, and the set *$ℛ=\left\{\left(I_{j},I_{j+1}\right):j\in \left(1,\ldots ,n-1\right\}\right\}$*of reference adjacencies, find a cancer genome I*_*α*(1)_*I*_*α*(2) _... *I*_*α*(*M*) _*satisfying:*

*1. for j *= 1, ..., *M *- 1 *either *$\left(I_{\alpha \left(j\right)},I_{\alpha \left(j+1\right)}\right)\in A$*or *$\left(I_{\alpha \left(j\right)},I_{\alpha \left(j+1\right)}\right)\in ℛ$.

*2. For k *= 1, ..., *n, the total number of indices j with α*(*j*) = *k or α*(*j*) = -*k is equal to c*_*k*_.

**Figure 1:**
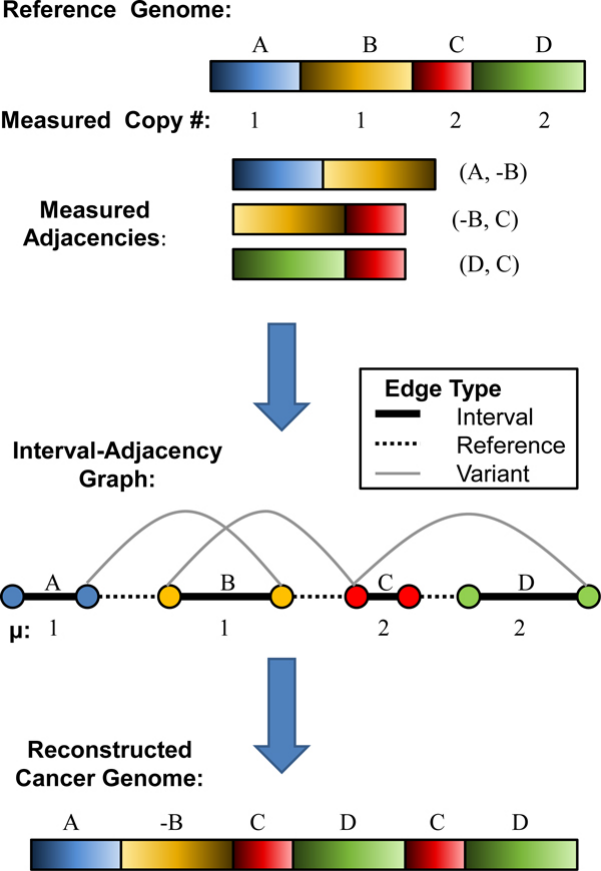
**Construction of the interval-adjacency graph**. Construction of the interval-adjacency graph. Paired-end sequencing data partitions a reference genome into intervals A, B, C, and D with associated copy numbers. These intervals and the measured adjacencies are used to build an interval-adjacency graph. Deriving the appropriate multiplicities on this graph results in an Eulerian tour which reconstructs a cancer genome consistent with the input data. Here, a possible reconstruction is A-B C D C D where -B indicates that the block is in the inverse orientation from the reference genome. Another possible reconstruction is A B C D C D which results from the assignment of multiplicity 0 to some variant edges.

To solve this problem, we introduce the *interval-adjacency *graph, which is derived from the interval vector **I **and cancer adjacencies  (Figure 1). The interval-adjacency graph *G *= (*V*, *E*) is an undirected graph with vertices *V *= {s_1_, *t*_1_, *s*_2_, *t*_2_, ..., *s*_*n*_, *t*_*n*_} and edges $E=E_{I}\cup E_{R}\cup E_{A}$. The set *E*_*I *_= {*e*_*I*_(*j*) = (*s*_*j*_, *t*_*j*_): *j *= 1, ..., *n*} of *interval edges *connect *s*_*j *_to *t*_*j *_for each *j*. The set *E*_*R *_of *reference edges *connect the ends of adjacent intervals in the reference genome; i.e. *E_R _*= {(*t*_*j*_, *s*_*j*+1_): *j *∈ {1, ..., *n *- 1}. The set $E_{A}$ of *variant edges *connect intervals that are adjacent in the cancer genome, but are not adjacent in the reference genome. These adjacencies are inferred from the set of discordant pairs. Every a∈A defines a variant edge. The interval, reference, and variant edges in the interval-adjacency graph are analogous to the gray, green, and black edges, respectively, in the breakpoint graph used in genome rearrangement analysis [25]. The interval-adjacency graph represents the set of possible adjacencies of intervals in the reference genome similar to how the gene order graph used in [28] contains possible gene orderings. Although, in that case the nodes of the graph represent genes and edges are gene adjacencies. Note that any *v *∈ *V *is incident to exactly one interval edge *I*_*j*_. Thus, we define *e*_*I*_(*v*) ∈ *E*_*I *_to be the interval edge containing vertex *v*, and define *e*_*I*_(*j*) ∈ *E*_*I *_to be the interval edge corresponding to interval *I*_*j*_. Similarly, we define *e*_*R*_(*v*) ⊆ *E*_*R *_to be the reference edge containing vertex *v*, if such an edge exists, and $E_{A}\left(v\right)\subseteq E_{A}$ to be the set of variant edges incident to vertex *v*.

Now if the data **I**, , and **c **are generated from an unknown cancer genome generated by a series of rearrangements, duplications and deletions that do not alter the chromosome ends (telomeres) *s*_1 _and *t*_*n*_, then the block organization of this cancer genome corresponds to an alternating path through *G *beginning at s_1 _and ending at *t*_*n *_that alternately traverses interval edges and non-interval edges (i.e. reference/variant edges), and where the number of times that each interval *I*_*j *_is traversed (in either direction) on the path is equal to *c*_*j *_(Figure 1). We require an alternating path since traversal of an interval edge is equivalent to selection of a block from the reference genome, and traversal of a reference/variant edge corresponds to a transition between blocks. Therefore, such an alternating path spells out a sequence of blocks from the reference genome. Formally, if we transform the interval-adjacency graph into a multigraph where the multiplicity of each edge equals the number of times it is traversed, then the multigraph has an Eulerian tour, as in the repeat graph, or deBruijn graph, in genome assembly algorithms [22,29].

Conversely, if we are given data **I**, , and **c **then we would like to infer an integer multiplicity *μ*(*e*) on each edge e such that an alternating Eulerian path from s_1 _to *t*_*n *_exists. We refer to *s*_1 _and *t*_*n *_as *telomeric vertices *and denote by $T=\left\{s_{1},t_{n}\right\}$ the set of telomeric vertices. Finding such an assignment of multiplicities can be formulated as an integer linear program (ILP). In particular, the restriction that the tour alternates between interval edges and non-interval (reference/variant edges) means that at each non-telomeric vertex *v*, the multiplicity of the interval edge *e*_*I*_(*v*) must equal the sum of the multiplicities of the reference edge *e*_*R*_(*v*) and variant edges $e_{A}\left(v\right)$. Telomeric vertices $T=\left\{s_{1},t_{n}\right\}$ are excluded from this requirement since by definition they are only incident to an interval edge, but not incident to any reference or variant edges. This constraint imposes the following *copy number balance *conditions on the multiplicities.

(1) $$\begin{array}{ll} \mu \left(e_{I}\left(v\right)\right)=\mu \left(e_{R}\left(v\right)\right) & +\underset{a:a\in E_{A\left(v\right)}}{\sum }\mu \left(a\right),\;\\ & \;\;\;\forall v\in V\backslash T.\;\\ & \; \end{array}$$

The following theorem follows directly from (1) and Kotzig's Theorem for alternating Eulerian paths [30] (see also [31]).

**Theorem 1**. *Given a connected interval-adjacency graph G *= (*V*, *E*)*, there exists a function μ: E* ↦ ℕ *satisfying the copy number balance conditions *(1) *if and only if there exists a multigraph G*_*μ *_= (*V*, *E*_*μ*_) *with edge multiplicities μ containing an alternating Eulerian Tour beginning at s*_1 _*and ending at t*_*n*_.

Finding such a function *μ *is the Eulerization problem and can be solved in polynomial time [24]. Applying the above result with the additional constraint *μ*(*e*_*I*_(*j*)) = *c*_*j *_for *j *= 1, ..., *n *provides an interval-adjacency multigraph that contains an alternating Eulerian tour, corresponding to a cancer genome consistent with the data I, , and c. In a later section, we extend Theorem 1 to the case of multiple chromosomes by finding a set of alternating tours.

In the case of perfect data, there is guaranteed to be a solution to the Eulerization problem: one such solution is the assignment of multiplicities that correspond to the cancer genome. However, there is no guarantee on the uniqueness of the solution, and other solutions - including solutions that do not use all variant edges - are possible. Figure 1 gives an example. In the case of perfect data we could require that all variant edges are assigned non-zero multiplicity, thus ensuring that all variant edges from the cancer genome are used. However, in the case of imperfect data addressed below, such constraints are not appropriate as we expect such data to contain missing and false adjacencies due to difficulties in inferring adjacencies (structural variants) from paired-end sequencing data.

### Copy number and adjacency genome reconstruction problem: imperfect data

The previous section considered the case where the intervals I and adjacencies  were derived from a cancer genome with no errors, and where the interval count vector c was known. Now we consider the situation that is presented by real data, where c is unknown and the adjacencies  may be incorrect (with missing adjacencies and/or false adjacencies). Instead of c, we are given a (paired) read depth vector r = (*r*_1_,..., *r*_*n*_*) *derived by the alignment of concordant paired reads to the reference genome. Each entry *rj *is the number of concordant pairs of reads that when aligned to the reference genome lie entirely within the interval *I*_*j*_. We use a probabilistic model to derive the most likely edge multiplicities *μ *in the interval-adjacency graph.

Specifically, let *L*_1_*, L*_2_, ..., *L*_*n *_be the lengths of intervals I = (*I*_1_, *I*_*2*_*, ..., I*_*n*_), and let $L_{R}=\sum_{i=1}^{n}L_{i}$be the length of the reference genome. Let $N=\sum_{i=1}^{n}r_{i}$ be the total number of concordant pairs that align within these intervals. Following the Lander-Waterman model, we assume that the reads are distributed uniformly on the genome, so that the number of reads that align to each interval follows the Poisson distribution with mean λ_*j *_equal to the expected number of reads that align to an interval *I*_*j*_. Of course, the Poisson distribution is an idealized assumption, and it has been shown that read depth is more accurately fit by a over-dispersed Poisson or negative binomial model [21,32]. Nevertheless, the Poisson assumption has proven useful for copy number variant detection [26], and thus we use the Poisson model here, postponing consideration of other distributions to later work. We assume that the length of the cancer genome is approximately equal to the length *L*_*r *_of the reference genome and *μ*_*j *_*= μ*(*e*_*I*_(*j*)) is the integer multiplicity assigned to the interval edge *I*_*j*_. In a genome without any rearrangements, we expect $\frac{NL_{j}}{L_{R}}$ concordant paired reads to align within interval *I*_*j *_(ignoring end effects). Since humans are diploid, we need to rescale this value to indicate the presence of two copies of interval *Ij*. Therefore, we introduce a variable *τ *that represents the expected number of copies of each interval in a non-rearranged sample. Given *τ*, the expected number of reads that align to an interval *I*_*j *_appearing *μ*_*j *_times in the genome is $\lambda_{j}\left(\frac{\mu_{j}}{\tau }\right)=\frac{NL_{j}}{L_{R}}\times \frac{\mu_{j}}{\tau }$. In general we set *τ *= 2, but we defer discussion of handling multiple chromosomes until the next section.

We define a convex optimization problem that finds the maximum likelihood assignment of multiplicities *μ*(*e*) to all edges *e *in the interval-adjacency graph G, subject to the copy number balance conditions discussed in the previous section. The likelihood function is the product over all interval edges *I*_*j *_of the Poisson probability of the observed number *r*_*j *_of concordant pairs that align within interval edge *I*_*j*_, which after taking the negative logarithm and removing constant terms gives us the (negative of) the likelihood function $L_{r}\left(\mu \right)=\sum_{j}\lambda_{j}\left(\frac{\mu_{j}}{\tau }\right)-r_{j}\text{log}\left(\lambda_{j}\left(\frac{\mu_{j}}{\tau }\right)\right)$. Thus, we have the following formulation.

(2) $$\underset{\mu }{\text{min}}L_{r}\left(\mu \right)=\overset{n}{\underset{j=1}{\sum }}\lambda_{j}\left(\frac{\mu_{j}}{\tau }\right)-r_{j}\text{log}\left(\lambda_{j}\left(\frac{\mu_{j}}{\tau }\right)\right)$$

subject to

(3) $$\begin{array}{c} \mu \left(e_{I}\left(v\right)\right)-\mu \left(e_{R}\left(v\right)\right)-\underset{a:a\in E_{A}\left(v\right)}{\sum }\mu \left(a\right)=0,\\ \forall v\in V\backslash T \end{array}$$

Setting ${ĉ}_{j}=\mu_{j}$ gives the most likely multiplicity for the interval *I*_*j *_in the cancer genome.

Note that [26] derives a similar formulation to predict germline copy number variants in human genomes, using a different construction based on bidirected graphs. Since human genomes are diploid, [26] add an additional source/sink vertex *σ *and add additional constraints that a flow of 2 be conserved across the graph. In contrast, most cancer genomes are aneuploid and might suffer deletions/duplications at the ends of chromosomes, this additional constraint is not applicable. We address this issue in the following section. [26] also show that their formulation reduces to a network flow problem that is solvable in polynomial time. The polynomial time result relies on two properties: (1) the objective function *L*_r_(*μ*) is separably convex; (2) the constraints are totally unimodular [33].

The interval-adjacency graph has a corresponding bidirected graph, and assignment of edge multiplicities in the interval-adjacency graph is equivalent to assignment of flow to the corresponding edges in the bidirected graph. Thus, the problem formulation in (2) above also reduces to a network flow problem that is solvable in polynomial time. In particular, for an interval-adjacency graph, we obtain a corresponding bidirected graph by adding orientation information to both ends of all edges in the original interval-adjacency graph. Specifically, for all interval edges (*s*_*j*_, *t*_*j*_*) *we assign a positive direction to the end at vertex *s*_*j *_and a negative direction to the end at vertex *t*_*j*_. For all reference edges (*t*_*j*_, s_*j*+1_) we assign a positive direction to the end at vertex *t*_*j *_and a negative direction to the end at vertex *s*_*j*_+1. For all the variant edges (*v*_1_*,v*_2_) we assign a positive direction for all *v *∈ {*v*_1_,*v*_2_} such that *v *is a vertex of the form *s*_*j*_, and a negative direction if *v *is a vertex of the form *t*_*j*_. We directly transfer all constraints on edge multiplicities. The problem formulation in (2) can now be equivalently described as a network flow problem on the corresponding bidirected graph since edge multiplicity assignment can be viewed as equivalent to flow assignment. Due to how we orient the bidirected edges, the copy number balance conditions from (1) are also equivalent to requiring that the amount of flow going into each vertex is equal to the flow exiting the vertex.

The formulation above addresses the fact that sequencing data does not directly give copy numbers of intervals, but rather yields read depth, which we use along with adjacencies to estimate copy number simultaneously across all intervals. However, another source of error in the data are incorrect and missing adjacencies in the set . Incorrect adjacencies will subdivide intervals and alter the read depths in each of these intervals. Because our likelihood function considers both read depth and adjacencies when determining edge multiplicities, our algorithm is somewhat robust to the presence of incorrect adjacencies. Incorrect adjacencies that do not alter the estimated copy numbers of intervals are likely not to be used (i.e. the adjacency will be assigned multiplicity *μ *= 0). Missing adjacencies will also affect the local structure of the interval-adjacency graph near the missing variant. In particular, all interval edges incident to the missing variant will be concatenated, and the corresponding variant edge will not be present. In most cases, we expect that the resulting reconstruction will simply not contain the missing adjacency. However, in other cases the missing adjacency may lead to additional errors in the reconstruction: for example the cases where the missing adjacency leads to large differences in the estimated copy number of the merged interval, or where the missing adjacencies overlaps with other variants. Our objective function (2) does not attempt to maximize the usage of variant edges, instead allowing the copy number estimates to determine whether variant edges are used are not. Defining an appropriate objective function that includes both copy number balance and scoring of variant edges is left for future work.

### Extensions: multiple chromosomes and telomere loss

We generalize the formulation above to handle two additional features of real data: (1) the reference and cancer genomes have multiple chromosomes, and (2) ends of chromosomes (telomeres) may be deleted in the generation of the cancer genome. First, to address the case of multiple chromosomes, we build a multichromosomal interval-adjacency graph *G *= (*V*, *E*) where the interval and reference edges are the union of interval and reference edges in the unichromosomal interval-adjacency graph, respectively. The variant edges $E_{A}$ are derived from the set  of adjacencies that connect intervals that are adjacent in the cancer genome, but not in the reference genome. These adjacencies are inferred from the discordant pairs, and now can include adjacencies between different chromosomes; e.g. those resulting from a translocation. The set  of telomeric vertices is the union of telomeric vertices of each chromosome, and consequently |T| is even. We now revise Theorem 1 to multi-chromosomal genomes, where we now decompose the interval-adjacency graph into a set of alternating tours.

**Theorem 2**. *Given an multichromosomal interval-adjacency graph G *= (*V*, *E*) *with telomeric vertices **, there exists a function μ*: *E *↦ ℕ *satisfying the copy number balance condition *(1) *for all *v∈V/T*if and only if there exists a multigraph G*_*μ *_= (*V*, *Eμ) with edge multiplicities μ containing a set of edge-disjoint alternating tours that each begin and end at vertices in**, and whose union is E*_*μ*_.

A second feature of cancer genome data is that telomeres of the reference genome may be lost. In this case, the set of telomeric vertices contains vertices other than the starts and ends of each chromosome of the reference genome. *De novo *telomere loss does not produce novel adjacencies in the cancer genome, and thus requires examining the read depth along the genome to find changes in concordant coverage, as used in read depth methods for copy number variant prediction [21]. Additionally, non-reciprocal translocations or breakage/fusion/bridge cycles produce novel adjacencies in the cancer genome and thus the drop in concordant coverage will be apparent over adjacent intervals in **I**. We use a heuristic which determines the relative ratio of concordant reads to interval length between intervals to determine these drops in concordant coverage, and if at least one such case is found, we add an additional vertex *σ *to the interval-adjacency graph and to the set  of telomeric vertices. We also add variant edges from *σ *to the incident interval edge of the loss.

## Results

We ran our PREGO algorithm on both simulated data and real sequencing data. We solve the convex optimization formulation in Equation (2) with CPLEX 12.1, using a piecewise linear approximation of the log term in the objective function, thus transforming the problem into an Integer Linear Program (ILP). Note, we use CPLEX rather than the efficient network flow algorithm discussed in a previous section as there is no good implementation of the later for bi-directed graphs.

### Ovarian sequencing data

We analyzed DNA sequencing data from 5 ovarian cancer genomes and matched normal samples that were sequenced as part of The Cancer Genome Atlas (TCGA) (Table 1 and Additional file 1). Each sample was sequenced at 30x coverage using Illumina paired end technology with read length of 36bp. We downloaded the BAM files containing aligned reads from TCGA Data portal, and used the GASV algorithm [14] to cluster discordant pairs from each sample and from the matched normal using only those paired reads with mapping quality ≥ 30 in the BAM file. We then removed any clusters of discordant pairs that contain paired reads from both the tumor sample and the matched normal. In this way, we focus on somatic rearrangements. We also require that the discordant clusters are: (1) at least 1Mb away from the centromeres as annotated in the UCSC Genome Browser; (2) that they have a minimum number (either 5 or 10 as indicated below) of supporting discordant pairs; (3) introduce intervals no smaller than 8Kb in the interval sequence **I**. Restricting the lengths of the intervals in **I **allows for a better estimation of read depth, which is obtained by counting the number of concordant pairs within each interval *I*_*j*_. We also restricted our analysis to the 22 autosomes. Table 1 gives the results of our algorithm when the cancer adjacencies  are restricted to those with at least 5 discordant pairs supporting each adjacency. The possible number of variants is quite large, and given the high rates of false positives with structural variant prediction [19,34] many of these are not likely to be real variants. Since we are lacking a set of validated structural variants for these ovarian cancer genomes, we examine in the next section features of the interval-adjacency graph that might help distinguish true variants.

**Table 1:** Ovarian dataset statistics

Dataset	ID	# Var Edges (Used)
OV1	TCGA-13-0890	771 (499)

OV2	TCGA-13-0723	562 (268)

OV3	TCGA-24-0980	311 (172)

OV4	TCGA-24-1103	340 (218)

OV5	TCGA-13-1411	389 (255)

Statistics of inferred interval-adjacency graphs for 5 ovarian genomes when a minimum of 5 discordant pairs are required to add a variant edge to the graph. A variant edge *e *is used if *μ*(*e*) > 0.

#### Reciprocal vs. non-reciprocal variants

**Figure 2:**
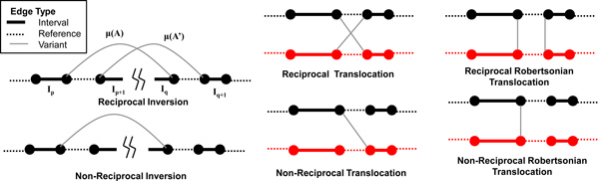
**Reciprocal and non-reciprocal variant edges**. Two classes of variant edges in the interval-adjacency graph. Reciprocal variants are pairs of variant edges incident to the same four interval edges (*I*_*p*_*, I*_*p*+1_, *I*_*q*_*, I*_*q+*1_), while non-reciprocal variants are the cases where only a single variant edge is incident to the four interval edges defined by the variant edge. A trivial reciprocal variant has equal inferred multiplicities: *μ*(*A*) *= μ*(*A'*), *μ*(*I*_*p*_) *= μ*(*I*_*p*+1_), and *μ*(*I*_*q*_) *= μ*(*I*_*q*+1_).

Each measured adjacency in A∈A represents the result of cutting the reference genome at two locations, resulting in four free "ends" of two pairs *I*_*p*_:*I*_*p+*1 _and *I*_*q*_:*I*_*q+*1 _of interval edges. Two of these ends are then pasted together in the cancer genome. In some cases, e.g. an inversion or a reciprocal translocation, there is a corresponding partner adjacency *A' *that joins together the other two free ends of the intervals. Note that the GASV algorithm [14] clusters discordant pairs to identify partner adjacencies, when present. Thus, we distinguish two types of variant edges in the interval-adjacency graph: non-reciprocal edges, and (pairs of) reciprocal edges. Figure 2 shows examples of both types of edges, including reciprocal and non-reciprocal inversions and translocations. Moreover, following the cytogenetic nomenclature, we distinguish two types of translocations: classical translocations that preserve the orientation of both chromosomes and Robertso-nian translocations that switch the orientation of one chromosome.

Thus, as a first step in evaluating the solutions produced by our algorithm, we examined the frequency with which reciprocal edges were used in the resulting interval-adjacency graph (i.e. the corresponding variant edge has inferred multiplicity > 0) versus the frequency with which non-reciprocal edges were used (Table 2). Note that reciprocal edges may be used in the following "trivial" way. If the inferred multiplicities on the two variant edges are both equal (i.e. *μ*(*A*) *= μ*(*A'*) *= k) *and the inferred multiplicities of each pair of interval edges surrounding the corresponding breakpoints are also equal (i.e. *μ*(*I*_*p*_) = *μ*(*I*_*p*+1_) and *μ*(*I*_*q*_) = *μ*(*I*_*q*+1_)) then the objective function (2) of the ILP is unchanged if one sets *μ*(*A*) = *μ*(*A*') = 0 and increases the edge multiplicity of the incident reference edges by *k*, thus removing the variant edges from the graph (Figure 2). We define reciprocal variant edges that satisfy this condition as *trivial *and those that do not satisfy this condition as *non-trivial*. Note that non-reciprocal variant edges have no equivalent trivial definition as altering the multiplicity assigned to a non-reciprocal variant edge would force a corresponding change in the multiplicity assigned the incident reference edges to maintain the copy number balance condition at the vertices of the variant edge. This change, however will cause the vertices at either end of the references edges to become unbalanced.

**Table 2:** Statistical tests for variant edges

Reciproc al vs. N on Reciprocal Variant Edges								
**Dataset**	**VariantType**	***R*(all)**	$\bar{R}$**(all)**	***R*(non-triv)**	$\bar{R}$**(non-triv)**	***NR***	$\bar{NR}$	**p-Val**

OV1	T	179	41	75	13	9	58	< 1E-15
OV1	I	46	20	16	12	2	29	3.46E-5
OV1	TO	210	46	70	16	9	38	2.79E-12

OV2	T	77	51	41	23	12	49	5.17E-7
OV2	I	21	15	9	5	10	21	0.057
OV2	TO	96	64	46	18	15	44	2.63E-7

OV3	T	61	13	19	3	6	30	2.111E-7
OV3	I	19	13	5	5	2	13	0.075
OV3	TO	58	26	22	8	7	28	1.92E-5

OV4	T	74	16	40	6	12	35	1.54E-9
OV4	I	10	0	2	0	3	12	0.073
OV4	TO	48	22	22	10	12	26	0.0036

OV5	T	93	19	29	7	8	37	2.30E-8
OV5	I	12	8	2	0	6	13	0.13
OV5	TO	82	26	22	8	7	34	2.29E-6

Results of Fisher's exact test showing that non-trivial reciprocal edges are more likely to be used (assigned a multiplicity *μ *> 0) in the interval-adjacency graph than non-reciprocal variant edges when a minimum of 5 discordant pairs is required to add a variant edge to the graph. Variant edges are classified as Inversion (I), Translocation (T), and Robertsonian Translocation (TO). Each variant edge is also classified as either reciprocal or not and by whether it is used (*μ *> 0) or not used (*μ *= 0). We report the number of edges of the following types: used reciprocal edges (R(all)), non used reciprocal edges ($\bar{R}$(all), used reciprocal non-trivial (*R*(non-triv)), not used reciprocal non-trivial ($\bar{R}$(non-triv)), used non-reciprocal (*NR*), and not used non-reciprocal ($\bar{NR}$).

We analyzed the output of our algorithm for reciprocal (non-trivial) edges and non-reciprocal variant edges. For each type of reciprocal variant (inversions, classical translocations and Robertsonian translocations) we tested whether there was an association between a variant edge being used vs. unused, and reciprocal vs. non-reciprocal, using Fisher's exact test. We find that in most cases there is a statistically significant association, with a larger fraction of (non-trivial) reciprocal variant edges being used than non-reciprocal variant edges (Table 2). We surmise that the observed significant association between reciprocal variants and their use in the solution obtained by our method is an indication that it may be easier to satisfy the copy number balance conditions for vertices associated with a reciprocal variant. In particular, we may only use a non-reciprocal variant if additionally the concordant coverage on the surrounding intervals is indicative of a possible change in copy number. In this respect, non-reciprocal variant edges that are used may represent structural variants whose signature is supported by both read depth and discordant read pairs.

#### Reconstructed variants

**Figure 3:**
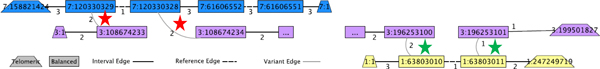
**Reciprocal translocations in OV5**. Examples of reciprocal Chr3/Chr7 (left) and Chr1/Chr3 (right) translocations in OV5. The Chr3/Chr7 translocation has the same multiplicity on the variant edges (red stars) as well as on the corresponding pairs of incident interval edges making it trivial. The Chr1/Chr3 translocation has different multiplicities on the variant edges (green stars) and is therefore non-trivial. In the Chr1/Chr3 translocation there is a single copy of Chr1 that does not use any variant edges, suggesting that only one copy of Chr1 is involved in the translocation, and that duplication of one of the translocated chromosomes occurs subsequent to the translocation.

In this section, we give several examples of reconstructed variants in the OV genomes. First, we show two cases of reciprocal translocations, one trivial and one non-trivial, demonstrating that in some cases we may infer possible ordering of rearrangements -for example a translocation preceding a duplication (Figure 3).

**Figure 4:**
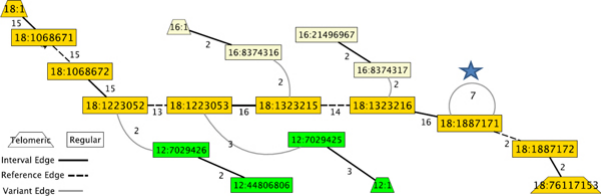
**BFB cycle on Chr18 in OV2**. Example of a Breakage/Fusion/Bridge Cycle on Chr18 in OV2. The first two Mb of Chr18 (starting in the upper left) is highly amplified, and this high multiplicity continues until the self-loop at Chr18:1887171 (blue star), which indicates an inverted repeat.

We also find subgraphs of the interval-adjacency graph that suggest particular mechanisms of aberrant DNA repair in cancer genomes. In particular, Figure 4 shows part of the interval-adjacency graph of the proximal arm of chromosome 18 in sample OV2. We identify highly amplified intervals that are incident to a loop variant edge that also has high multiplicity. Loops in the interval-adjacency graph are indication of inverted duplications, a signature of breakage/fusion/bridge cycles, a known source of genome instability in cancer genomes [35]. Oncogenes YES1 and TYMS appear in this amplified region, and both have been implicated in ovarian cancer [36,37].

**Figure 5:**
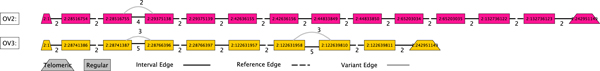
**Tandem duplications on Chr2 in OV2 and OV3**. Tandem duplications found on Chr2 in OV2 and OV3. OV2 has a single site of tandem duplication, while OV3 has two sites of tandem duplication. Note that the region duplicated in OV2 is much larger than the region duplicated on OV3, and the duplicated region in OV2 contains several cancer associated genes including PLB1, PPP1CB, ALK [39-41].

We also find tandem duplications on Chr2 of both OV2 and OV3 (Figure 5). Recently, a tandem duplication signature was reported in SNP data from Ovarian TCGA samples as well as in a pair of cell lines [38]. In particular, the cell line data included tandem duplications on Chr2. In the interval-adjacency graph, the location of these tandem duplications on the homologs of Chr2 are ambiguous. For example, OV2 has two copies of the variant edge, which may be one tandem duplication present on both copies of Chr2 or two tandem duplications present on one copy of Chr2. OV3 has two different locations where tandem duplications occur, one of which is within 2Mb of the duplicated region on OV2. All three of these tandem duplications occur with 4Mb of a duplication reported in [38] and one duplicated region in OV2 includes several cancer associated genes including PLB1, PPP1CB, ALK [39-41].

### Simulated data

We tested our algorithm on simulated data to determine how robust the reconstructed interval-adjacency graphs are to various errors in the input data. Errors in the input data arise from a number of sources, and we studied the effect of two types of errors on the performance of a simulated sequence: sample contamination and read depth estimation error. We begin by constructing a cancer genome *C *= *I*_*α*(1)_*I*_*α*(2) _... *I*_*α*(*M*) _consisting of 200 novel adjacencies: 100 homozygous deletions and 100 heterozygous deletions distributed over 22 autosomes (similar to the ovarian cancer genomes we analyzed in the previous section). The lengths of the deletions are sampled from a normal distribution with mean 10Kb and standard deviation 1Kb. From *C *we identify the sequence of intervals **I**. We introduce 50 additional "false" adjacencies, where each false adjacency simply partitions an interval in **I **into three subintervals and adds a corresponding false deletion adjacency to the set . We then simulate 30x physical coverage of paired-end sequencing by sampling uniformly from *C *the starting positions of intervals, called *read-intervals*. We sample the length of these intervals from a normal distribution with mean 200 and standard deviation 10. We compute the resulting read depth *r*_*j *_for each interval *I*_*j*_.

Tumor samples are often a mixture of cells from the tumor itself and cells from non-cancerous cells. To model this type of error, we sample some proportion *ρ *of the read-intervals from the corresponding reference genome (i.e. the sequence of intervals *I*_1_*I*_2 _*... I*_*n*_), and sample (1 -- *ρ) *of the read-intervals from the cancer genome *C*. Additional noise in the read depth estimation occurs due to experimental error (such as sequencing errors and alignment errors due to repetitive sequences in the reference genome) when estimating *r*_*j*_. Thus, we add Gaussian noise to each *r*_*j *_drawn from $N\left(0,\phi r_{j}\right)$. We use *ϕr*_*j *_rather than a single variance parameter to adjust the noise model for intervals with different read depths.

**Figure 6:**
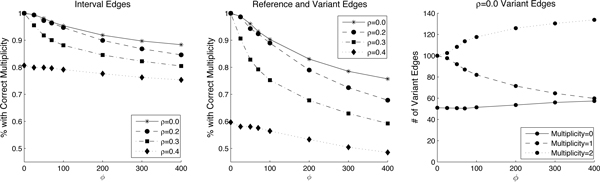
**Simulations**. Effect of sample contamination and read depth estimation errors on a simulated cancer genome. *ϕ *is a scaling factor for the variance; for example *ϕ *= 400 means that the noise model has a standard deviation 20 times *r*_*j *_for interval *I*_*j*_. We show the average percent of interval edges (left) and reference and variant edges (middle) correctly estimated over 10 trials. (Right) At *ρ *= 0, as *ϕ *increases most of the errors result from variant edges moving from the correct multiplicity of 1 (heterozygous deletion) to a multiplicity of 2 (homozygous deletion).

We ran our algorithm on the simulated datasets with error parameters *ρ *and *ϕ *and counted the number of edges in the interval-adjacency graph where the predicted multiplicity is the same as the correct multiplicity and averaged the results over 10 trials (Figure 6). The percent of correct edges drops by at most by 40%. Most of the errors made as the read depth variance *ϕ *increases are that heterozygous deletions are incorrectly called either homozygous no deletion (Figure 6).

## Discussion

The PREGO algorithm presented here combines copy number and adjacency information from paired-end sequencing data to infer cancer genome organization. However, the algorithm does not consider all the issues involved in real cancer sequencing data. In particular, we assume that structural variants can be identified by mapping of discordant paired reads, but this is difficult for structural variants in repetitive regions of the human genome [15,17]. Thus, there may be missing or incorrect adjacencies in the data. Similarly, estimates of read depth are difficult to obtain in repetitive regions [21]. While some of these issues may be addressed computationally, the more difficult cases will require longer reads and/or longer fragments for paired reads.

Beyond the issues with data quality are limitations on the inferred organization. While we derive multiplicities on the edges using adjacency and copy number data, we do not resolve the resulting paths through the interval-adjacency graph, except in simple cases. In many datasets, there will be many such paths and therefore many reconstructions of the cancer genome that are consistent with the data. Even the solution for the estimated edge multiplicities may not be unique. Resolving such longer paths requires additional information about connections between consecutive adjacencies, and such information is generally not available unless the distance between consecutive adjacencies is within the length of a read/fragment. In addition, the interval-adjacency graph does not contain allele-specific information about copy number variants, as considered in other work [35]. Finally, we assume that a cancer sample contains a single genome, when in fact most cancer samples contain DNA from a mixture of tumor cells, each with potentially different somatic mutations. It is possible that some of this intratumor heterogeneity could be resolved computationally. Alternatively, DNA sequencing of single cells, or smaller pools of cells, will minimize these effects.

An additional area of investigation is to infer the temporal history of rearrangements. In the case of copy-neutral rearrangements, inferences can be made using parsimony models such as Hannenhalli-Pevzner theory [42]. This approach has previously been used in cancer genome analysis [13]. Models have also been introduced to infer orders of mutations in cases where there is interaction between duplications and rearrangements [43] and duplications and single-nucleotide mutations [35,44].

## Conclusions

We formulated the Copy Number and Adjacency Genome Reconstruction Problem of reconstructing a rearranged cancer genome and developed an efficient algorithm, called Paired-end Reconstruction of Genome Organization (PREGO), for a particular instance of this problem. We designed an optimization problem on the interval-adjacency graph, which is related to the breakpoint graph used in genome rearrangement studies. We applied our algorithm to 5 ovarian cancer genomes sequenced as part of The Cancer Genome Atlas (TCGA) and reconstruct structural variants in these genomes. We analyzed the patterns of reciprocal vs. non-reciprocal rearrangements, and identified rearrangements consistent with known mechanisms of duplication such as tandem duplications and breakage/fusion/bridge cycles.

## List of abbreviations used

TCGA: The Cancer Genome Atlas; B/F/B: breakage/fusion/bridge; ILP: integer linear program

## Competing interests

The authors declare that they have no competing interests.

## Authors' contributions

BJR, LO and SA conceived of the project. BJR supervised the work. LO implemented the algorithm and performed experiments. AR and RD aided in performing experiments. LO, AR and BRJ wrote the manuscript. All authors read and approved the manuscript.

## Supplementary Material

Additional file 1**Figures of the interval-adjacency graph derived for all 5 ovarian genomes analyzed when cancer adjacencies ****are restricted to those with at least 10 discordant pairs supporting each adjacency**.Click here for file

## References

[B1] Albertson DG Collins C McCormick F Gray JW Chromosome aberrations in solid tumors Nat Genet 2003 34 4 369 376 10.1038/ng1215 12923544

[B2] Druker BJ Talpaz M Resta DJ Peng B Buchdunger E Ford JM Lydon NB Kantarjian H Capdeville R Ohno Jones S Sawyers CL Efficacy and Safety of a Specific Inhibitor of the BCR-ABL Tyrosine Kinase in Chronic Myeloid Leukemia New England Journal of Medicine 2001 344 14 1031 1037 10.1056/NEJM200104053441401 11287972

[B3] Kauraniemi P Hautaniemi S Autio R Astola J Monni O Elkahloun A Kallioniemi A Effects of Herceptin treatment on global gene expression patterns in HER2-amplified and nonamplified breast cancer cell lines Oncogene 2004 23 4 1010 3 http://www.ncbi.nlm.nih.gov/pubmed/14647448 10.1038/sj.onc.1207200 14647448

[B4] Raphael B Volik S Yu P Wu C Huang G Linar-dopoulou E Trask B Waldman F Costello J Pienta K Mills G Bajsarowicz K Kobayashi Y Sridharan S Paris P Tao Q Aerni S Brown R Bashir A Gray J Cheng J de Jong P Nefedov M Ried T Padilla-Nash H Collins C A sequence-based survey of the complex structural organization of tumor genomes Genome Biol 2008 9 R59 10.1186/gb-2008-9-3-r59 18364049 PMC2397511

[B5] Greenman C Stephens P Smith R Dalgliesh GL Hunter C Bignell G Davies H Teague J Butler A Stevens C Edkins S O'Meara S Vastrik I Schmidt EE Avis T Barthorpe S Bhamra G Buck G Choudhury B Clements J Cole J Dicks E Forbes S Gray K Hal-liday K Harrison R Hills K Hinton J Jenkinson A Jones D Menzies A Mironenko T Perry J Raine K Richardson D Shepherd R Small A Tofts C Varian J Webb T West S Widaa S Yates A Cahill DP Louis DN Goldstraw P Nicholson AG Brasseur F Looijenga L Weber BL Chiew YE DeFazio A Greaves MF Green AR Campbell P Birney E Easton DF Chenevix-Trench G Tan MH Khoo SK Teh BT Yuen ST Leung SY Wooster R Futreal PA Stratton MR Patterns of somatic mutation in human cancer genomes Nature 2007 446 7132 153 8 10.1038/nature05610 17344846 PMC2712719

[B6] Stephens PJ Greenman CD Fu B Yang F Bignell GR Mudie LJ Pleasance ED Lau KW Beare D Stebbings LA McLaren S Lin ML McBride DJ Varela I Nik-Zainal S Leroy C Jia M Menzies A Butler AP Teague JW Quail MA Burton J Swerdlow H Carter NP Mors-berger LA Iacobuzio-Donahue C Follows GA Green AR Flanagan AM Stratton MR Futreal PA Campbell PJ Massive genomic rearrangement acquired in a single catastrophic event during cancer development Cell 2011 144 27 40 10.1016/j.cell.2010.11.055 21215367 PMC3065307

[B7] Meyerson M Gabriel S Getz G Advances in understanding cancer genomes through second-generation sequencing Nat Rev Genet 2010 11 685 696 10.1038/nrg2841 20847746

[B8] Mardis ER Wilson RK Cancer genome sequencing: a review Hum Mol Genet 2009 18 R2 R163 R168 10.1093/hmg/ddp396 19808792 PMC2758710

[B9] Schatz MC Delcher AL Salzberg SL Assembly of large genomes using second-generation sequencing Genome Res 2010 20 1165 1173 10.1101/gr.101360.109 20508146 PMC2928494

[B10] Tuzun E Sharp AJ Bailey JA Kaul R Morrison VA Pertz LM Haugen E Hayden H Albertson D Pinkel D Olson MV Eichler EE Fine-scale structural variation of the human genome Nat Genet 2005 37 7 727 732 10.1038/ng1562 15895083

[B11] Korbel JO Urban AE Affourtit JP Godwin B Grubert F Simons JF Kim PM Palejev D Carriero NJ Du L Taillon BE Chen Z Tanzer A Saunders ACE Chi J Yang F Carter NP Hurles ME Weissman SM Harkins TT Gerstein MB Egholm M Snyder M Paired-end mapping reveals extensive structural variation in the human genome Science 2007 318 5849 420 426 10.1126/science.1149504 17901297 PMC2674581

[B12] Volik S Zhao S Chin K Brebner JH Herndon DR Tao Q Kowbel D Huang G Lapuk A Kuo WL Magrane G De Jong P Gray JW Collins C End-sequence profiling: sequence-based analysis of aberrant genomes Proceedings of the National Academy of Sciences of the United States of America 2003 100 13 7696 701 http://www.pnas.org/cgi/content/abstract/100/13/7696 10.1073/pnas.1232418100 12788976 PMC164650

[B13] Raphael BJ Volik S Collins C Pevzner PA Reconstructing tumor genome architectures Bioinformatics 2003 19 Suppl 2 ii162 ii171 http://bioinformatics.oxfordjournals.org/cgi/content/abstract/19/suppl\_2/ii162 10.1093/bioinformatics/btg1074 14534186

[B14] Sindi S Helman E Bashir A Raphael BJ A geometric approach for classification and comparison of structural variants Bioinformatics (Oxford, England) 2009 25 12 i222 30 http://www.pubmedcentral.nih.gov/articlerender.fcgi?artid=2687962\&tool=pmcentrez\&rendertype=abstract 10.1093/bioinformatics/btp208 PMC2687962 19477992

[B15] Hormozdiari F Alkan C Eichler EE Sahinalp SC Combinatorial algorithms for structural variation detection in high-throughput sequenced genomes Genome Res 2009 19 7 1270 1278 10.1101/gr.088633.108 19447966 PMC2704429

[B16] Chen K Wallis JW McLellan MD Larson DE Kalicki JM Pohl CS McGrath SD Wendl MC Zhang Q Locke DP Shi X Fulton RS Ley TJ Wilson RK Ding L Mardis ER BreakDancer: an algorithm for high-resolution mapping of genomic structural variation Nat Methods 2009 6 677 681 10.1038/nmeth.1363 19668202 PMC3661775

[B17] Quinlan AR Clark RA Sokolova S Leibowitz ML Zhang Y Hurles ME Mell JC Hall IM Genome-wide mapping and assembly of structural variant breakpoints in the mouse genome Genome Res 2010 20 623 635 10.1101/gr.102970.109 20308636 PMC2860164

[B18] Xi R Kim TM Park PJ Detecting structural variations in the human genome using next generation sequencing Briefings in functional genomics 2010 9 5-6 405 15 http://bfg.oxfordjournals.org/content/9/5-6/405.full 10.1093/bfgp/elq025 21216738 PMC3080742

[B19] Medvedev P Stanciu M Brudno M Computational methods for discovering structural variation with next-generation sequencing Nature methods 2009 6 11 Suppl S13 20 19844226 10.1038/nmeth.1374

[B20] Chiang DY Getz G Jaffe DB O'Kelly MJT Zhao X Carter SL Russ C Nusbaum C Meyerson M Lander ES High-resolution mapping of copy-number alterations with massively parallel sequencing Nature methods 2009 6 99 103 10.1038/nmeth.1276 19043412 PMC2630795

[B21] Yoon S Xuan Z Makarov V Ye K Sebat J Sensitive and accurate detection of copy number variants using read depth of coverage Genome Res 2009 19 1586 1592 10.1101/gr.092981.109 19657104 PMC2752127

[B22] Pevzner PA Tang H Waterman MS An Eulerian path approach to DNA fragment assembly Proc Natl Acad Sci USA 2001 98 9748 9753 10.1073/pnas.171285098 11504945 PMC55524

[B23] Pevzner PA Tang H Fragment assembly with double-barreled data Bioinformatics 2001 17 Suppl 1 S225 S233 http://bioinformatics.oxfordjournals.org/cgi/content/abstract/17/suppl\_1/S225 10.1093/bioinformatics/17.suppl_1.S225 11473013

[B24] Medvedev P Brudno M Maximum likelihood genome assembly J Comput Biol 2009 16 1101 1116 10.1089/cmb.2009.0047 19645596 PMC3154397

[B25] Alekseyev MA Pevzner PA Breakpoint graphs and ancestral genome reconstructions Genome research 2009 19 5 943 57 http://genome.cshlp.org/cgi/content/abstract/19/5/943 10.1101/gr.082784.108 19218533 PMC2675983

[B26] Medvedev P Fiume M Dzamba M Smith T Brudno M Detecting copy number variation with mated short reads Genome Res 2010 20 11 1613 1622 10.1101/gr.106344.110 20805290 PMC2963824

[B27] Campbell PJ Stephens PJ Pleasance ED O'Meara S Li H Santarius T Stebbings LA Leroy C Edkins S Hardy C Teague JW Menzies A Goodhead I Turner DJ Clee CM Quail MA Cox A Brown C Durbin R Hurles ME Edwards PAW Bignell GR Stratton MR Futreal PA Identification of somatically acquired rearrangements in cancer using genome-wide massively parallel paired-end sequencing Nature genetics 2008 40 6 722 9 10.1038/ng.128 18438408 PMC2705838

[B28] Wittler R Manňuch J Patterson M Stoye J Consistency of sequence-based gene clusters J Comput Biol 2011 18 9 1023 1039 10.1089/cmb.2011.0083 21899413

[B29] Pevzner PA Tang H Tesler G De novo repeat classification and fragment assembly Genome Res 2004 14 1786 1796 10.1101/gr.2395204 15342561 PMC515325

[B30] Kotzig A Moves without forbidden transitions in a graph Mathematica Slovaca 1968 18 76 80

[B31] Pevzner P DNA physical mapping and alternating Eulerian cycles in colored graphs Algorithmica 1995 13 77 105 10.1007/BF01188582

[B32] Bentley DR Accurate whole human genome sequencing using reversible terminator chemistry Nature 2008 456 53 59 10.1038/nature07517 18987734 PMC2581791

[B33] Hochbaum D Shanthikumar J Convex separable optimization is not much harder than linear optimization Journal of the ACM (JACM) 1990 37 4 843 862 10.1145/96559.96597

[B34] Mills RE Mapping copy number variation by population-scale genome sequencing Nature 2011 470 59 65 10.1038/nature09708 21293372 PMC3077050

[B35] Greenman CD Pleasance ED Newman S Yang F Fu B Nik-Zainal S Jones D Lau KW Carter N Edwards PAW Futreal PA Stratton MR Campbell PJ Estimation of rearrangement phylogeny for cancer genomes Genome Res 2012 22 2 346 361 10.1101/gr.118414.110 21994251 PMC3266042

[B36] Steinhardt AA Gayyed MF Klein AP Dong J Maitra A Pan D Montgomery EA Anders RA Expression of Yes-associated protein in common solid tumors Hum Pathol 2008 39 1582 1589 10.1016/j.humpath.2008.04.012 18703216 PMC2720436

[B37] Kelemen LE Genetic variation in TYMS in the one-carbon transfer pathway is associated with ovarian carcinoma types in the Ovarian Cancer Association Consortium Cancer Epidemiol Biomarkers Prev 2010 19 1822 1830 10.1158/1055-9965.EPI-09-1317 20570913 PMC3013232

[B38] Ng CK Cooke SL Howe K Newman S Xian J Temple J Batty EM Pole JC Langdon SP Edwards PA Bren-ton JD The role of tandem duplicator phenotype in tumour evolution in high-grade serous ovarian cancer J Pathol 2011 10.1002/path.3980 22183581

[B39] Moestue SA Borgan E Huuse EM Lindholm EM Sitter B Børresen-Dale AL Engebraaten O Maelandsmo GM Gribbestad IS Distinct choline metabolic profiles are associated with differences in gene expression for basal-like and luminal-like breast cancer xenograft models BMC Cancer 2010 10 433 10.1186/1471-2407-10-433 20716336 PMC2931488

[B40] Takakura S Kohno T Manda R Okamoto A Tanaka T Yokota J Genetic alterations and expression of the protein phosphatase 1 genes in human cancers Int J Oncol 2001 18 4 817 824 11251179 10.3892/ijo.18.4.817

[B41] Jung Y Kim P Jung Y Keum J Kim SN Choi YS Do IG Lee J Choi SJ Kim S Lee JE Kim J Lee S Kim J Discovery of ALK-PTPN3 gene fusion from human non-small cell lung carcinoma cell line using next generation RNA sequencing Genes Chromosomes Cancer 2012 10.1002/gcc.21945 22334442

[B42] Hannenhalli S Pevzner PA Transforming men into mice (polynomial algorithm for genomic distance problem) Proc th Annual Symp Foundations of Computer Science 1995 581 592

[B43] Raphael BJ Pevzner PA Reconstructing tumor am-plisomes Bioinformatics 2004 20 Suppl 1 i265 i273 10.1093/bioinformatics/bth931 15262808

[B44] Durinck S Ho C Wang NJ Liao W Jakkula LR Collisson EA Pons J Chan SW Lam ET Chu C Park K Hong Sw Hur JS Huh N Neuhaus IM Yu SS Grekin RC Mauro TM Cleaver JE Kwok PY LeBoit PE Getz G Cibulskis K Aster JC Huang H Purdom E Li J Bolund L Arron ST Gray JW Spellman PT Cho RJ Temporal Dissection of Tu-morigenesis in Primary Cancers Cancer Discovery 2011 http://cancerdiscovery.aacrjournals.org/content/early/2011/06/23/2159-8290.CD-11-0028.abstract 10.1158/2159-8290.CD-11-0028 PMC3187561 21984974

