# Supplementary material for: Reconstructing cancer genomes from paired-end sequencing data
Source: BMC Bioinformatics. 2012 Apr 19;13(Suppl 6):S10. doi: 10.1186/1471-2105-13-S6-S10 (PMC3358655; doi:10.1186/1471-2105-13-S6-S10)
Supplement: Additional file 1 — Figures of the interval-adjacency graph derived for all 5 ovarian genomes analyzed when cancer adjacencies are restricted to those with at least 10 discordant pairs supporting each adjacency. [file 1471-2105-13-S6-S10-S1.pdf]

Supplemental Figures for Reconstructing Cancer Genomes from Paired-end Sequencing Data

Layla Oesper[*]
layla@cs.brown.edu

Anna Ritz[*]
aritz@cs.brown.edu

Sarah J. Aerni[†]
saerni@stanford.edu

Ryan Drebin[*]
ryan_drebin@brown.edu

Benjamin J. Raphael[*,‡]
braphael@cs.brown.edu

[*]Department of Computer Science, Brown University, Providence, RI.
[†]BioMedical Informatics Program, Stanford University, Stanford, CA.
[‡]Center for Computational Molecular Biology, Brown University, Providence, RI.

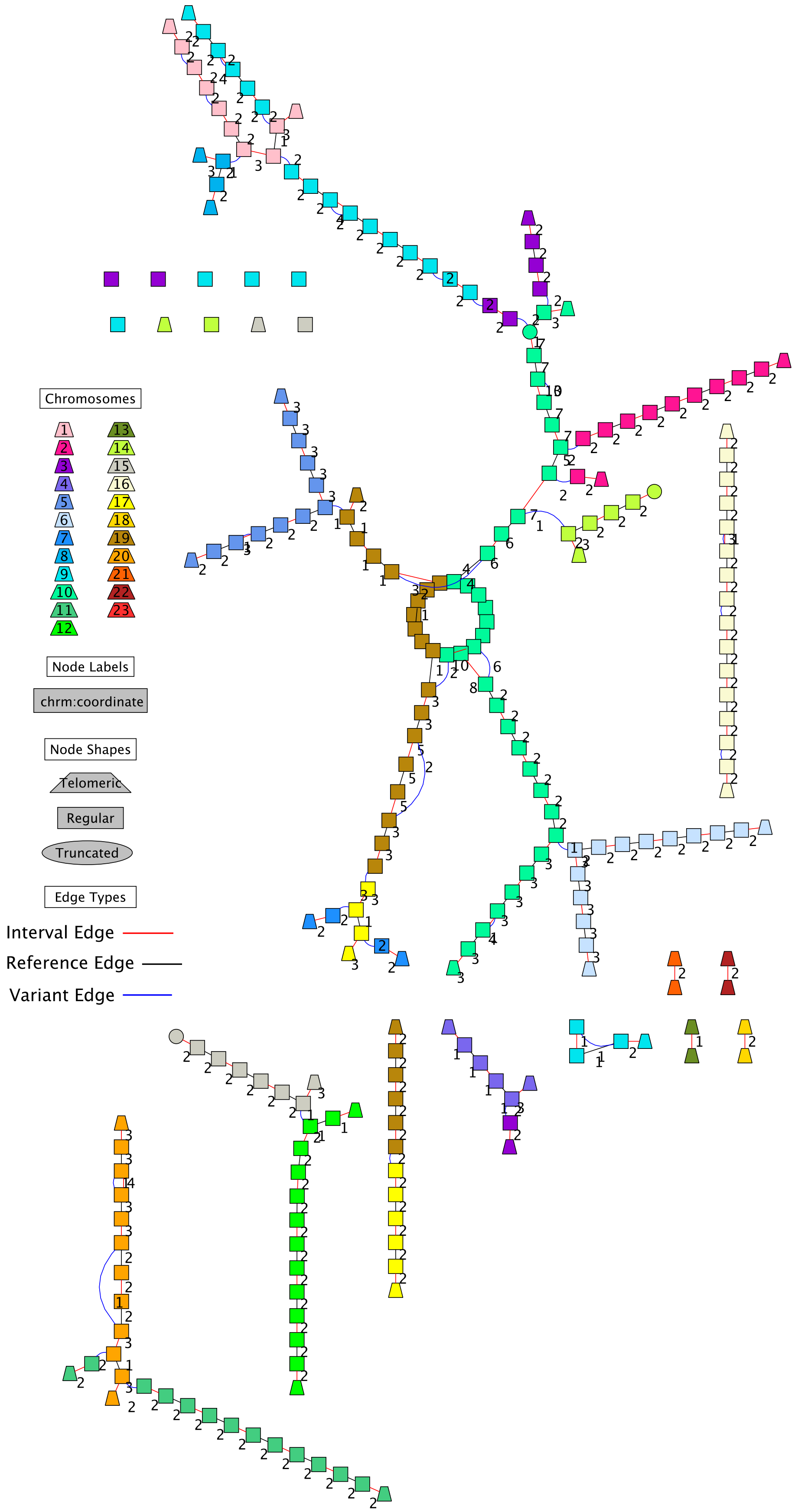

Figure 1: Cancer Genome Reconstruction for OV1 when a minimum of 10 discordant pairs are required to add a variant edge to the graph.

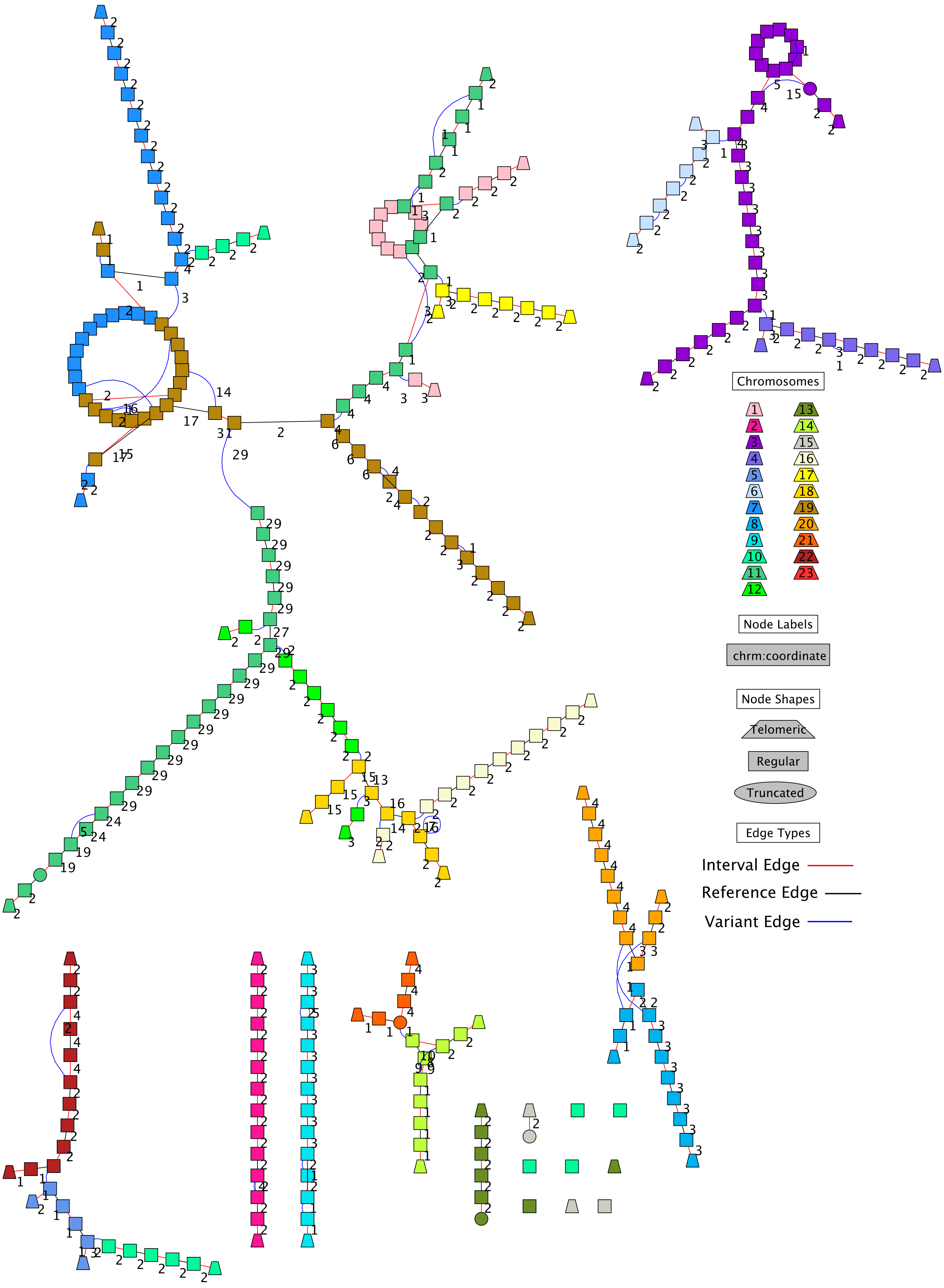

Figure 2: Cancer Genome Reconstruction for OV2 when a minimum of 10 discordant pairs are required to add a variant edge to the graph.

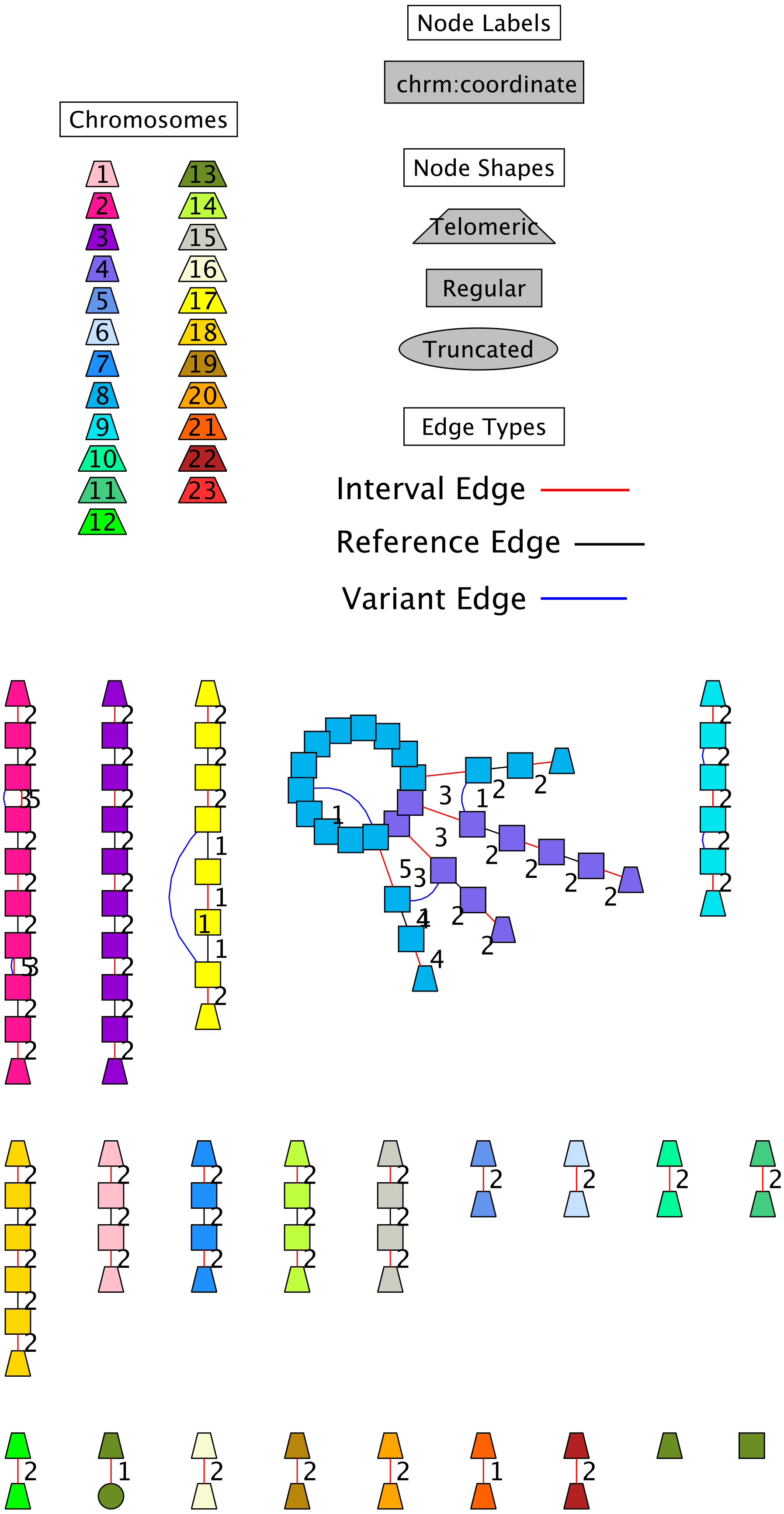

Figure 3: Cancer Genome Reconstruction for OV3 when a minimum of 10 discordant pairs are required to add a variant edge to the graph.

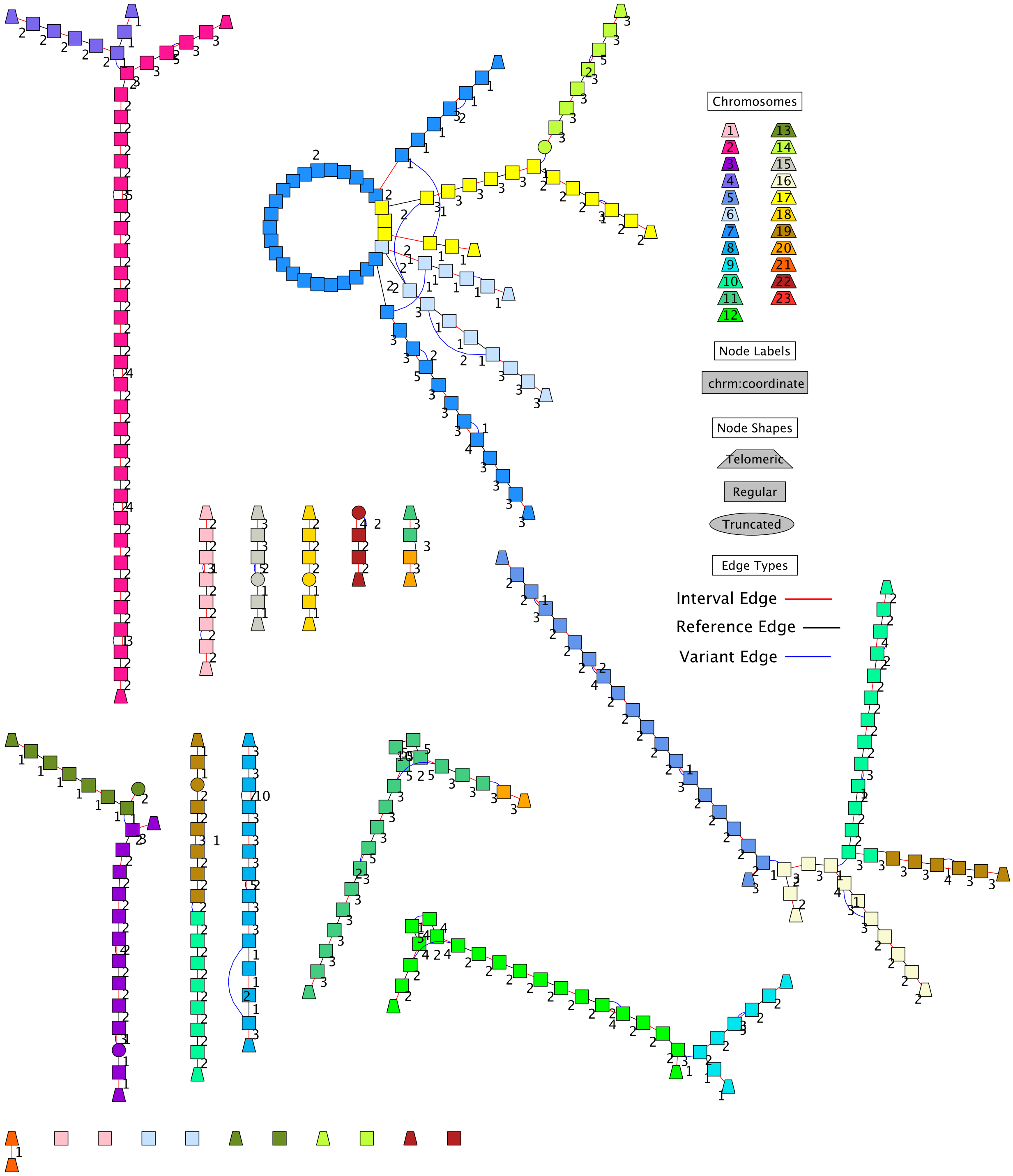

Figure 4: Cancer Genome Reconstruction for OV4 when a minimum of 10 discordant pairs are required to add a variant edge to the graph.

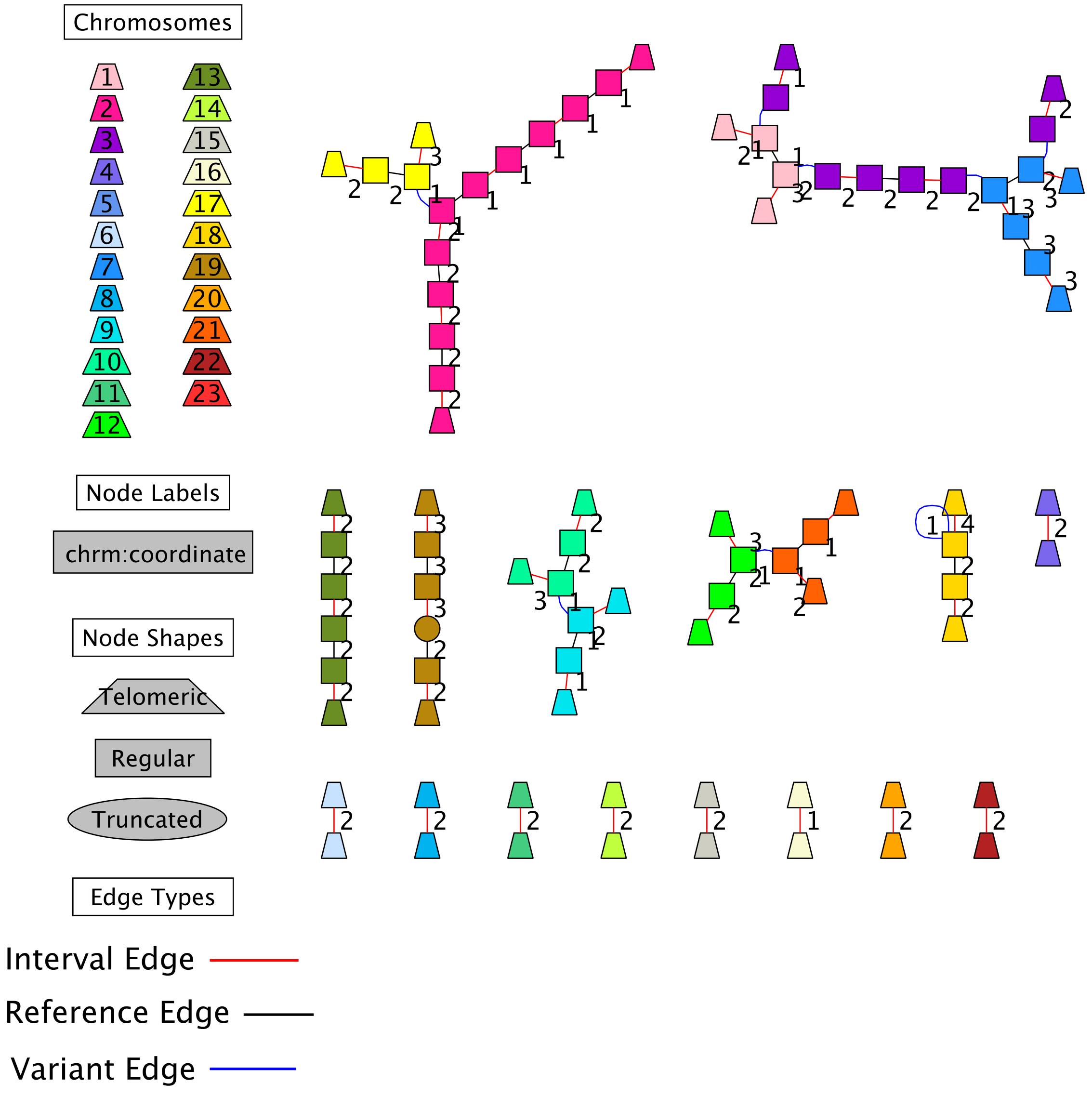

Figure 5: Cancer Genome Reconstruction for OV5 when a minimum of 10 discordant pairs are required to add a variant edge to the graph.
